# ASSESSING RACIAL DISPARITIES IN UTILIZATION OF HEALTHCARE SERVICES, 2010-2020

**DOI:** 10.1093/geroni/igae098.3793

**Published:** 2024-12-31

**Authors:** Yanjun Dong, Kun Wang, Victoria Rizzo

**Affiliations:** SUNY Albany, Albany, New York, United States; College of Arts & Sciences News, The University of Alabama at Birmingham, Alabama, United States; University at Albany, State University of New York, University at Albany, State University of New York, New York, United States

## Abstract

**Objectives:**

Little is known about the intersectional effects of race, gender, income, and education on healthcare service utilization among BIPOC (Black, Indigenous, and People of Color) older adults. This study fills this gap by analyzing longitudinal data of older adults in the U.S. from 2010 to 2020.

**Methods:**

Using six waves of longitudinal data from the Rand Health and Retirement Study (n= 26,304), this study employed random-effect Poisson and logistic models to examine disparities across seven healthcare services, focusing on the interactions of BIPOC with gender, education, and income.

**Results:**

The findings show disparities in healthcare utilization, with BIPOC older adults consistently reporting lower utilization across different types of services, including hospital stays, prescription drug use, outpatient surgery, home healthcare, physician visits, and dental care. For the interactions, the results indicate that, compared with others, low-income BIPOC older adults are less likely to access prescription drugs (β = -1.10, p < 0.01) but more likely to use home healthcare (β = 0.52, p < 0.05) and physician services (β = 0.75, p < 0.05). BIPOC women were less likely to utilize nursing home services (β = -0.84, p < 0.05).

**Discussion:**

This study underscores the effects of socioeconomic factors and systemic inequities on healthcare utilization. Policy implications include the need for targeted interventions to reduce financial barriers, culturally competent care models, and enhanced health literacy programs. The findings highlight the urgent need for systemic changes to create an equitable healthcare system that addresses the needs of marginalized populations.

